# Lecture on Cactus Grandiflorus

**Published:** 1885-09

**Authors:** J. T. Kent

**Affiliations:** St. Louis


### LECTURE ON CACTUS GRANDIFLORUS.

PROFESSOR J. T. KENT, M. D., ST. LOUIS.

One of the most prominent symptoms is a sensation of constriction. The heart feels as if grasped by an iron hand. The throat or the uterus may have the same constrictive feeling. Also about limbs, throat, head, and chest. (Lilium-tig. has a squeezing sensation about the heart, but not so violent as Cactus). This sensation is attended with prickling and numbness of the body. Spasms of the circular fibres, of heart and uterus in particular. It seems capable of producing contractions in the skin and wherever there are orifices. There may be sharp, sticking pains, with horrible constriction about the spincters. (Apis, Phos., Bry., and Lach. have a sensation of suffocation about the heart. Rhus. and Digitalis have an aching numbness.) Violent dysmenorrhœa with constriction. (Cham., Puls., Bell., and Valer. are often given for this, but more frequently Morphine by mongrels.) Generally vertigo, with heart symptoms. (For this Puls. is more often the remedy.) Cactus produces congestion of the brain, blood-shot eyes, coma, suffocation, and fullness of the veins, which symptoms are all found in heart disease. Pulsation in the temple (Bell.) as if the artery would burst. Headache, especially on the right side (Bell.), worse from lying down; sometimes attended with nose-bleed—a violent rush of bright, red blood. Face blue, pale, flushed, covered with cold sweat. (The blueness is very similar to Carbo-animalis.) The constriction of the throat excites a constant desire to swallow, which does not relieve, like Lach. Constriction of the œsophagus, must drink large quantities to force water into the stomach (œsophagismus); constrictive feeling at the scrobiculus cordis, extending to the hypochondria, impeding breathing. Sensation of a belt around the abdomen. Pulsation generally is in harmony with Cactus (but Puls. has it more prominently). (Ant. t. has throbbing in the pit of the stomach, relieved by vomiting). Engorgement of the liver, acute or chronic, from heart disease. This engorgement is secondary to heart disease. Feeling as if a cord was tightly bound around the lower part of the chest, marking out the attachments of the diaphragm. Sharp pains shooting through the diaphragm up into the stomach. Spasmodic constriction of the anus, preventing stool, and sometimes so violent that it becomes very painful. *Urine* straw-colored, with much burning and constriction of the neck of the bladder. Menses too soon, black and pitch-like; menstruation, with constrictive spasms of the uterus; pain agonizing and worse in the evening; flow scanty and ceasing when lying down (menses always cease at night—Bovista). Many of the spasms of Cactus are brought on when lying down. Voice low and hoarse from constriction of the chest. Oppression of breathing on going up-stairs. Uneasiness, as if an iron band prevented normal breathing; cannot lie on the left side. Pulse throbbing, tense, and hard. Dyspnœa is an important symptom; endocardial murmurs: If you have organic disease of the heart you may not cure it, but no matter what the symptoms are, if they call for Cactus give it, it might do considerable good. Cactus is a deep-acting medicine, and is capable of affecting an organic change. Irregularity of the heart's action, at times slow, great irritation of the heart; enlarged left ventricle. Sensation of constriction in the middle of the sternum. Rheumatism of the muscles of the chest. Dropsy of the hands and feet consequent on heart disease. Chill not relieved by covering; returning at the same hour each day—11 A. M. and 11 P. M. The chills, pain, and constrictions occur regularly each day at 11 P. M. Intermittent fever with congestion to the head. Retention of urine. The entire body feels as if caged, each wire being twisted tighter and tighter. One prover thought that the walls were narrowing down around him.

